# Enhancing Meat Emulsion Quality and Storage Stability During Refrigeration Using Thyme and Oregano Essential Oil Nanoparticles

**DOI:** 10.3390/foods14061076

**Published:** 2025-03-20

**Authors:** Syed A. Hussain, Sarfaraz A. Wani, Sheikh Rafeh, Sheikh Adil, Asif H. Sofi, Heba I. Ghamry, Manzoor Wani

**Affiliations:** 1 Division of Livestock Products Technology, FVSc &AH, Sher-e-Kashmir University of Agricultural Science and Technology (Kashmir), Jammu & Kashmir 190006, India; dr.arshidsyed@gmail.com (S.A.H.);; 2 Division of Livestock Production and Management, FVSc &AH, Sher-e-Kashmir University of Agricultural Science and Technology (Kashmir), Jammu & Kashmir 190006, India; 3 Nutrition and Food Science, Department of Biology, College of Science, King Khalid University, Abha 61421, Saudi Arabia

**Keywords:** thyme, oregano, essential oil, nanotechnology, meat emulsion, storage stability

## Abstract

The ability to efficiently store raw emulsion and market it as a ready-to-cook convenience meat product would be extremely advantageous to society and the global meat business. With this innovation, consumers may easily make a range of fresh emulsion-based meat products, saving time and labour. The current study was thus designed with the goal of improving the quality and storage stability of meat emulsions by using chitosan-based thyme (*Thymus vulgaris*) and oregano (*Origanum vulgare*) essential oil nanoparticles as natural preservatives. The treatments included the following: T0—control; T1—emulsion added with chitosan nanoparticles @ 500 ppm; T2—emulsion added with thyme essential oil nanoparticles @ 500 ppm; T3—emulsion added with oregano essential oil nanoparticles @ 500 ppm; and T4—positive control added with synthetic additive butylated hydroxytoluene @ 200 ppm. TBARS (Thiobarbituric acid reactive substances) values revealed that T2 and T3 exhibited greater oxidative stability throughout storage. Protein carbonyl levels increased at a slower rate during storage in nano-treated essential oil groups. DPPH (2, 2 diphenyl-1-picryl hydrazyl) and FRAP (Ferric Reducing Anti-Oxidant Power) values decreased significantly (*p* < 0.05) during storage, with T3 having the strongest anti-oxidant activity. T2 and T3 had consistently greater texture values than the other groups. T2 and T3 demonstrated lower values for microbiological parameters, particularly on day 7 and 15. The storage stability period of emulsion was 3 days for T0 and T4, while as it was 6 days for T1 and 9 days for T2 and T3. T2 and T3 showed higher sensory scores, affirming their superior sensory appeal to other treatments. In conclusion, the essential oil nanoparticle treatments resulted in better quality and storage stability of meat emulsions during aerobic refrigerated storage.

## 1. Introduction

Emulsion-based meat products form an important and major category of meat products, which add variety to our food basket in the form of meat balls, sausages, loaves, patties, nuggets, etc. Apart from this, emulsion formation helps in the profitable utilisation of tough meat and a reduction in the product cost by the inclusion of edible by-products like organ meat, etc., in the formulation, as well as improving the yield and quality of the finished products. However, emulsion preparation is a tedious process involving specialised equipment and technical knowledge in addition to being labour-intensive and time-consuming [1]. Thus, it would be in the best interest of society as well as the global meat industry if raw emulsion could be preserved and marketed as a ready-to-cook convenience meat product, suitably packed in appropriate packages with instructions for further cooking. This would aid in quick product development by avoiding the wastage of time and labour in emulsion preparation, besides avoiding post-process contamination and thermal processing disadvantages. However, as compared to intact meat, ground and emulsion-based meat products are highly susceptible to oxidative degradation owing to the increased addition of fat as well as the physical disruption of muscle and cell integrity, which diminishes endogenous protective anti-oxidant systems and increases the surface area of meat for the action of molecular oxygen and other intrinsic pro-oxidants [2]. The enhanced surface area and increased manipulation of meat during emulsion formation also amplify the chances of microbial degradation manifold. Furthermore, the thermal processing of the meat accelerates the oxidative problem by the rapid generation of free radicals and destruction of endogenous anti-oxidant systems, making cooked meat more susceptible to oxidative degradation than raw meat [3].

In order to curb these degradative processes, a variety of synthetic preservatives like nitrates and nitrites, polyphosphates, monosodium glutamate (MSG), butylated hydroxyanisole (BHA), butylated hydroxytoluene (BHT), tertiary butyl hydroquinone (TBHQ), propyl, octyl and dodecyl gallates, etc., have been used in the meat industry over the years. However, the toxicological and environmental concerns associated with the use of these synthetic preservatives and additives have exacerbated the growing legal and consumer demand for wholesome and minimally processed foods free from synthetic preservatives. With this in view, the industry has shifted toward a trend of using natural preservatives to limit the oxidative and microbial spoilage of meat during cold storage [4]. Among various types of natural preservatives, essential oils (EOs) of aromatic and medicinal herbs have been widely accepted owing to their strong antimicrobial and anti-oxidant activities along with their ephemeral and biodegradable nature [5]. Amongst various herbs, thyme and oregano occupy an important place. These are aromatic plants of the Mediterranean flora commonly used for medicinal purposes. Thyme (*Thymus vulgaris*) essential oil possesses antimicrobial, antifungal, anti-oxidant and antiviral properties [6]. Oregano (*Origanum vulgare*) essential oil is also known to possess antimicrobial, antifungal and anti-oxidant activities, along with anti-mutagenic and anti-carcinogenic effects [7]. Moreover, these essential oils and their main constituents, phenolic compounds, viz. carvacrol and thymol, do not have any mutagenic effect on humans [8] and are categorised as GRAS (Generally Recognized as Safe), thus making them suitable candidates for meat preservation [9].

However, despite the advantages offered by essential oils, there are certain limitations associated with their use in food systems, like interactions with food matrix components, viz. fat, protein and starch; low solubility in the aqueous phase; the alteration of sensorial characteristics of foodstuff at high concentration; sensitivity to oxygen, light and heat during processing, utilisation and storage; and highly volatile character [10]. Here, nanotechnology provides an efficient alternative to overcome these issues and involves the generation and utilisation of materials at a nanometre scale with particle sizes below 100 nm [11]. By nanoencapsulation, the enclosed wall preserves the encapsulated/loaded substances (essential oils, phenolic compounds, proteins, enzymes, vitamins, minerals and others) against thermal and chemical degradation and increases their stability in the corresponding environment [10], with the improved regulation of their release at the active physiological site, thereby controlling their delivery at the desired time and site [12]. Nanoencapsulation also serves to decrease the volatility of essential oils, masking their unpleasant odours and tastes [13,14]. It increases their solubility in the aqueous phase, promoting their bioavailability and functional activity, and improves the characteristics of the final food product [15].

Keeping in view the aforementioned facts, the proposed work was envisaged with the objective of assessing the effect of the direct addition of thyme and oregano essential oil nanoparticles on the quality and storage stability of meat emulsion under aerobic refrigerated storage conditions.

## 2. Materials and Methods

### 2.1. Subjects

This study was reviewed and approved by the Sher-e-Kashmir University of Agricultural Science and Technology (Kashmir) under approval no. Au/Acad/PG/PF/2022/5921-22, and informed consent was obtained from each subject prior to their participation in the study.

### 2.2. Materials

Hind leg portions from freshly dressed male sheep carcasses (preferably 12 to 18 months of age) were procured from the local market. The lean meat obtained after hot boning was used for the preparation of the emulsion. Animal fat used in the experiments was preferably obtained from the carcasses of the same kill.

The essential oils used in the experiments were organic and of food-grade quality. Thyme essential oil (*Thymus vulgaris*) was imported from Zongle Therapeutics LLC, Norcross, GA, USA; GCIAOCP-USDA Organic-certified. Wild oregano essential oil (*Origanum vulgare*) was imported from Aromavita Ltd. S. Kyprianou, 1, Limassol, Cyprus, Greece. Chitosan (shrimp shell origin), with a ≥75% degree of deacetylation, was procured from HiMedia Laboratories Pvt. Ltd., Mumbai, India. All the chemicals (analytical or molecular grade) were obtained from standard firms.

The packaging materials were procured from standard firms. Low-density polyethylene (LDPE) pouches (200 gauge/2 mil/51 μm thickness) were used for aerobic packaging, and multilayered laminated pouches (590 gauge/5.9 mil/150 μm thickness) were used for the vacuum packaging of the emulsion samples.

### 2.3. Preparation of Nanoparticles

The chitosan nanoparticles (CNPs), thyme essential oil nanoparticles (TNPs) and oregano essential oil nanoparticles (ONPs) were prepared according to the standardised method of “Emulsification and Ionic gelation’’ as described by Keawchaoon and Yoksan [16]. Chitosan solution [1.5% (*w*/*v*)] was prepared by dissolving chitosan in glacial acetic acid 1% (*v*/*v*) on a hotplate magnetic stirrer for 45 min at 60 °C. After complete dissolution, the solution was centrifuged at 5000 rpm for 10 min to clear any heterogeneous impurities in the solution. Tween 80 (0.16%) was added into the chitosan solution, and the pH of the solution was adjusted to 4.2 by NaOH solution (2 N). The mixture was stirred at 50 °C for 30 min to achieve a homogeneous solution. Then, the essential oils were added drop-wise into chitosan solutions to achieve the required chitosan/EO ratio of 1:0.75 during simultaneous homogenisation by a homogeniser (WiseTis HG-15D, Wisenberg, Switzerland) at a speed of 13,000 rpm for 10 min under an ice-bath condition for the purpose of achieving an oil-in-water emulsion. Finally, sodium tripolyphosphate (TPP) was added drop-wise to induce the ionic gelation of chitosan under constant magnetic stirring (1000 rpm/25 °C). The agitation was continued for another 45 min to allow the complete crosslinking of chitosan with tripolyphosphate ions, resulting in the spontaneous formation of nanoparticles. No essential oil was added for formation of nascent chitosan nanoparticles (CNPs). The nanoparticle suspensions were finally frozen in a deep freezer overnight before being subjected to freeze drying (Lyovapor L-200, Flawil, Switzerland). The freeze-dried powdered samples were packaged in air-tight containers and stored at −18 °C until further analysis and use.

### 2.4. Morphological Assessment of Nanoparticles

The morphological properties of different nanoparticles were evaluated through field-emission scanning electron microscopy (FESEM) (ZEISS Gemini SEM 500, 8203017193, Gaithersburg, UK). The samples were fixed on aluminium stubs using adhesive carbon tape, mounted on the grid and coated with a thin film of gold (sputter coater, Model SC7620) before FESEM images were taken at 100 K × magnification.

### 2.5. Emulsion Preparation

Manual pounding as practised in the traditional method was replaced using machines like a meat mincer and bowl chopper for the controlled, efficient and hygienic production of emulsion. The machine processes as standardised at the Division of Livestock Products Technology, FVSc and AH, SKUAST-Kashmir, was adopted for emulsion preparation, and the emulsion was prepared as per the method of Hussain et al. [17] with slight modifications.

The details of various treatments are given in Table 1. The treated emulsions (T0, T1, T2, T3 and T4) were packaged aerobically in LDPE pouches and stored at refrigeration temperature (4 ± 1 °C). The samples were analysed for 15 days at regular intervals of 3 days (days 0, 3, 6, 9, 12, 15) for most of the parameters, except for emulsion stability, colour and texture, which were analysed at the beginning, middle and end.

### 2.6. Physico-Chemical Qualities

#### 2.6.1. pH

The pH of emulsion samples was determined by the method of Trout et al. [18] by using a combination glass electrode digital pH meter (Model EE-011, Tanco Laboratory Equipments Ltd., New Delhi, India). A total of 10 g of the samples (in duplicate) per treatment per trial was homogenised with the help of a Polytron homogenizer (PT-MR-2100, Filderstadt, Germany) for about a minute in 100 mL of freshly distilled water. The pH was recorded by immersing the electrode directly into the meat suspension.

#### 2.6.2. Peroxide Value

The peroxide value was determined by monitoring the iodine liberated from Potassium Iodide (KI) by lipid peroxides as described by Akhter et al. [19] with some modifications. The peroxide value (PV) was calculated and is expressed in milliequivalents of active oxygen per kilogramme (meq O_2_/kg meat) using the following equation:

PV (meq/kg sample) = [(V × N)/W] × 1000

where

V= volume (mL) of sodium thiosulphate used;
N = normality of sodium thiosulphate;
W = weight of sample (g).

#### 2.6.3. Thiobarbituric Acid Reactive Substances (TBARS)

The TBARS value was determined according to the method of Serrano et al. [20]. First, 2 g of the sample from each emulsion batch in duplicate was homogenised in 8 mL of 5% trichloroacetic acid (TCA) with the help of a Polytron homogenizer (PT-MR-2100, Germany). The homogenised sample was filtered through ashless Whatman filter paper No.1, and the filtrate was adjusted to 10 mL with 5% TCA. An aliquot of 5 mL of filtrate was mixed with 5 mL of 20 mM aqueous TBA reagent and placed in the dark for 20 h at room temperature. The absorbance was measured at a fixed wavelength of 532 nm using a UV/VIS Spectrophotometer (Hitachi, UV-Spectrophotometer U-1800, Tokyo, Japan). The malonaldehyde (MDA) concentration was obtained by means of a standard curve using standard solutions of gradually increasing concentrations (0.0005 to 0.002 mM, R^2^ = 0.994) of 1,1,3,3-tetraethoxypropane, and the results were expressed as mg MDA/Kg of sample.

#### 2.6.4. Protein Carbonyls

Protein carbonyls were assayed as hydrazone derivatives by reacting proteins with 2, 4-dinitrophenylhydrazine (DNPH) according to the method of Srinivasan and Hultin [21]. The difference in absorbance at 370 nm between HC1-treated aliquots (control) and DNPH-treated aliquots (sample) was taken as a measure of reacted carbonyl groups using a molar extinction coefficient of 2.2 × 10^4^ M^−1^ cm^−1^ for protein hydrazones, as described by Levine et al. [22], using the following equation:

Protein carbonyls (nmol/mg protein) = [(A_370_ sample − A_370_ control)/22,000] × 10^6^

#### 2.6.5. 2,2 Diphenyl-1-picryl Hydrazyl Activity

The anti-oxidant activity of meat emulsion extracts based on DPPH (2, 2 diphenyl-1-picryl hydrazyl) radicals was analysed following the method given by Brand-Williams et al. [23]. The radical scavenging activity was measured using the following formula:

Radical scavenging activity (%) = [(A_blank_ − A_sample_)/A_blank_] × 100

#### 2.6.6. Ferric Reducing Anti-Oxidant Power

The FRAP (Ferric Reducing Anti-Oxidant Power) assay was performed according to the procedure described by Benzie and Strain [24]. The method is based on the reduction of a Ferric–TPTZ (tripyridyltriazine) complex (colourless) to a Ferrous–TPTZ complex (bluish colour) at low pH, which is monitored by measuring the absorption change at 593 nm.

#### 2.6.7. Texture Profile Analysis

The texture profile of the emulsion samples was analysed using the back extrusion method on a TA-XT plus texture analyser from Stable Micro Systems (Vienna Court, Godalming, UK) equipped with a 5 kg load cell, as described by Ghirro et al. [25], with certain modifications. The results were obtained using Exponent lite software V6 1.18.0 Version proprietary to Stable Micro Systems.

#### 2.6.8. Colour Analysis

A colour analysis of the emulsion samples was carried out by the CIELAB system by assessing L*, a* and b* values as defined by the Commission Internationale de l’Eclairage (CIE, International Commission on Illumination). The colour difference (ΔE) was calculated by using the following equation: (ΔE) = (ΔL*^2^ + Δa*^2^ + Δb*^2^)^1/2^. The colour attributes were determined using a colorimeter (Sucolor-Precision Colorimeter, Model SC-10, SN:5003062, 3NH Technology Co., Ltd., Shenzhen, China) with a standard illumination of D65, a viewing aperture of 4 mm and a 10^0^ standard observer angle. Three surface measurements were taken on each side of the packed emulsion sample, giving a total of 2 average values per sample.

### 2.7. Microbiological Quality

The meat emulsion samples were subjected to microbiological analysis for the total plate count, coliform count, yeast and mould count, psychrotrophic plate count and anaerobic plate count as per the method described by APHA [26].

A total of 10 g of sample was aseptically weighed and transferred to a pre-sterilised mortar. A 90 mL volume of sterile 0.1% peptone water was added to it, and samples were homogenised for 2 min using a sterile pestle for uniform dispersion, yielding a 10^−1^ dilution. For obtaining a 10^−2^ dilution, 1 mL of this diluted solution was transferred to a test tube containing 9 mL of sterile 0.1% peptone water. The procedure was repeated by taking 1 mL from the 10^−2^ dilution to another tube with 9 mL sterilised 0.1% peptone water for obtaining a 10^−3^ dilution. The procedure was continued up to the achievement of the required dilution coefficient. All procedures were performed under a Laminar Air Flow near a flame, creating practically efficient sterilised conditions. Plate Count Agar (procured from Himedia laboratories Pvt. Ltd., Mumbai, India) for total plate count, Violet Red Bile Agar (VRBA) for coliform count, Potato Dextrose Agar (procured from Hi-media Laboratories Pvt. Limited, Mumbai, India) for yeast and mould count, and Plate Count Agar (procured from Himedia laboratories Pvt. Ltd., Mumbai) for psychrotrophic plate count were utilised.

### 2.8. Emulsion Stability

The emulsion stability of the samples was determined as per the method of Horita et al. [27] with some modifications. A total of 35 g of the meat emulsion samples (in duplicate) per treatment per trial was placed in polypropylene tubes and cooked in a water bath at 80 °C for 30 min. The tubes were then taken out and left to stand upside down for 45 min to release the exudates in pre-weighed moisture cups. The total amount of fluid released (TFR) was weighed and expressed in a percentage of the sample weight as an index of emulsion stability.

### 2.9. Sensory Evaluation

For conducting the sensory evaluation, the raw emulsion samples were presented to a group of no less than 7 semi-trained panellists consisting of the scientists and post-graduate students of the Division of Livestock Products Technology, FVSc and AH, SKUAST-Kashmir. The samples were evaluated for various sensory parameters, viz. odour, colour, texture and overall acceptability, as per a modified score card, which was prepared on the basis of scores as adopted by Zhou et al. [28] based on a 5-point descriptive scale, where 5 = extremely desirable and 1 = extremely undesirable. A score of 3.5 corresponding to desirable sensory characteristics of the products was taken as the minimum score for the acceptability of the product.

### 2.10. Statistical Analysis

The whole set of experiments was repeated thrice (three trials) for the consistency of the results, and samples for each parameter were drawn in duplicate for analysis, leading to a total of 6 observations (*n* = 6); for sensory attributes, seven panellists analysed the samples, leading to a total of 21 observations (*n* = 21). The data generated from the experiments were pooled and subjected to statistical analysis using analysis of variance (ANOVA), and results are expressed as means with standard error. The means were compared using Duncan’s multiple range test (DMRT), with a significance level of 0.05, using SPSS package (SPSS 20.0 for Windows, SPSS Inc., Chicago, IL, USA).

## 3. Results and Discussion

### 3.1. Morphological Properties of Nanoparticles

**Figure 1:**
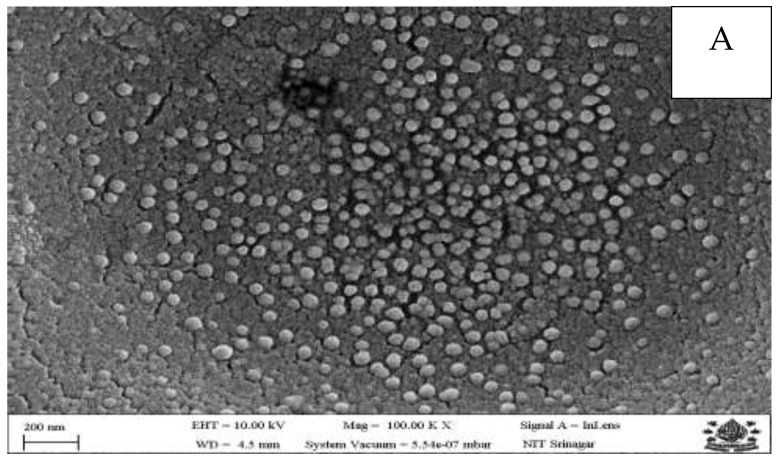

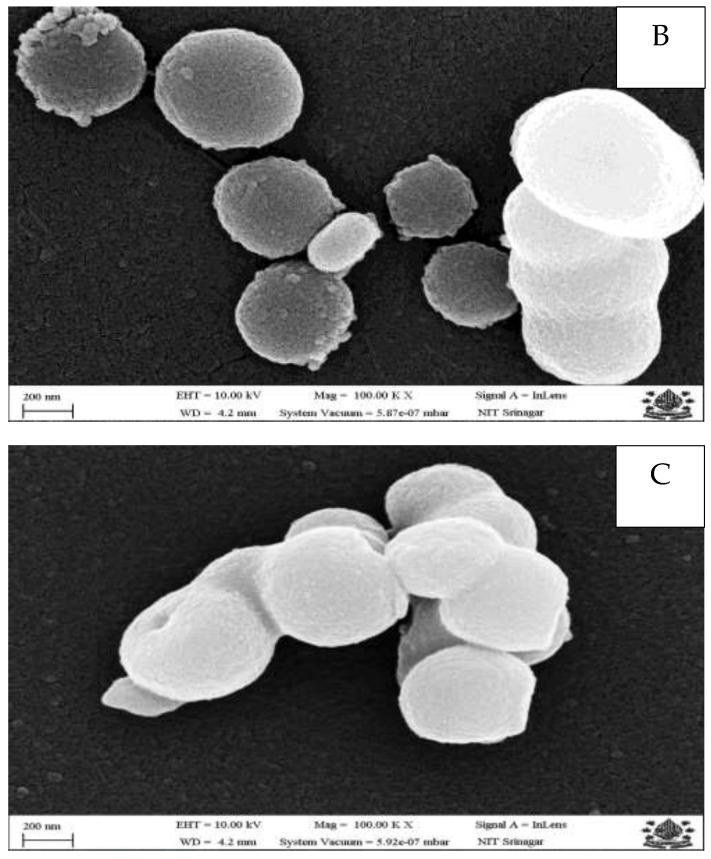
FESEM images of different nanoparticles, (**A**): chitosan nanoparticles; (**B**): thyme essential oil nanoparticles; (**C**): oregano essential oil nanoparticles.

The FESEM images of different nanoparticles (Figure 1) demonstrated uniformly distributed spherical nanosized particles, signifying the efficiency of the process adopted for nanoparticle preparation. Furthermore, the essential oil-loaded nanoparticles displayed aggregations in some places. Similar findings for essential oil-loaded nanoparticles were reported previously [29]. The different nanoparticles demonstrate sizes in the range of 13–30 nm.

### 3.2. Physico-Chemical Qualities

#### 3.2.1. pH

The pH of all the treatments showed an increasing trend with the storage period, with the increase being significant (*p* < 0.05) in T0 at each successive storage interval (Table 2). However, at each storage interval, the pH of T2 and T3 was significantly (*p* < 0.05) lower than T1, which was significantly (*p* < 0.05) lower than T0 and T4. The accumulation of volatile bases like ammonia, biogenic amines and trimethylamine formed during the protein hydrolysis and breakdown of amino acids by microorganisms or endogenous enzymes is what most likely causes a pH rise in samples [30] and is in good agreement with previous reports in meat and meat products undergoing nanoencapsulated essential oil treatments [31]. The significantly (*p* < 0.05) lower pH values in T1, T2 and T3 may be attributed to the antibacterial effect of chitosan in CNPs and the synergistic antibacterial effect of chitosan and essential oils in TNPs and ONPs [32].

#### 3.2.2. Peroxide Value

Hydroperoxides are primary lipid oxidation products. Therefore, the measurement of the peroxide value provides a useful indication of the extent of lipid oxidation. The peroxide value (PV) of all the treatments including the control (T0) first significantly (*p* < 0.05) increased up to day 9 of storage and thereafter showed a significant (*p* < 0.05) decrease (Table 3), which could be due to the faster decomposition of hydoperoxides (into secondary oxidation products like aldehydes) than their production [33]. Within the storage intervals, the lower PV in T2 and T3 might be due to the synergistic anti-oxidant effect of chitosan and essential oils. Thyme and oregano essential oils owe their anti-oxidant activity to their phenolic compounds, mainly thymol and carvacrol, which are amongst the best known phenolic compounds employed in meat preservation, along with other minor constituents like p-cymene and **γ**-terpinene [34]. It has been proven that phenolic compounds can retard the formation of metabolites of the auto-oxidation process, thus inhibiting/delaying the onset of lipid oxidation [35]. The anti-oxidant activity of chitosan is due to its residual amino groups which react with volatile aldehydes like malonaldehyde generated from the decomposition of fats, leading to the formation of a stable fluorosphere, and also due to its potency in chelating ferrous ions [36]. In addition, nanoencapsulation serves to prevent essential oil evaporation and decomposition during storage and protect the bioactive compounds against the adverse effects of oxygen and temperature, offering the sustained release of essential oils along with a nanometric size, which increases the surface area of the essential oils, enhancing their overall anti-oxidant activities [37].

#### 3.2.3. TBARS

Secondary oxidation products, particularly malonaldehyde, are mainly responsible for the off-flavour and oxidative rancidity of meat and meat products. The TBARS values followed an increasing trend, increasing significantly (*p* < 0.05) with the storage period, with T2 and T3 exhibiting lower values as compared to other treatments and the control (Table 3). From day 9 to day 12, the increase in TBARS was abrupt, coinciding with the decrease in peroxide values, which could be due to the degradation of hydroperoxides to secondary oxidation products like malonaldehyde (MDA), as has also been reported by other authors [31]. According to Sheard et al. [38], MDA concentrations higher than 0.5 mg/kg are threshold values for rancidity perception by consumers, whereas according to Esmaeili et al. [39], meat products with MDA concentrations higher than 1mg/kg emanate rancid odours. The threshold value of 0.5 mg/kg was reached very early in T0 (3rd day), followed by T1 (6th day) and T4 (9th day), and much later in nanoparticle treatments, viz. T2 and T3 (12th day).

#### 3.2.4. Protein Carbonyls

Protein carbonylation is considered one of the most remarkable chemical changes during protein oxidation and is an important factor that can cause changes in meat quality [40]. Lipid and protein oxidations have a profound impact on each other, which is further signified by the results of this study. The carbonyl content of all the treatments significantly (*p* < 0.05) increased as storage progressed (Table 3). However, the carbonyl contents of T2 and T3 were lower than all the other treatments including the control on all storage days for the reasons already discussed above. It has been reported that the carbonyl content of non-oxidised tissue is generally <1 nmol/mg protein [41]. This threshold was surpassed in T0 and T1 on the 3rd day and in other treatments later on, thus emphasising the anti-oxidant effect of T2, T3 and T4. Our results of the significantly (*p* < 0.05) lower carbonyl contents of T2 and T3 are in concurrence with other studies, where essential oil/essential oil nanoparticle treatments reported a significantly (*p* < 0.05) lower carbonyl content as compared to the control and even BHT [31].

#### 3.2.5. DPPH and FRAP

The total anti-oxidant activities of different meat emulsion treatments were evaluated by both DPPH and FRAP methods (Table 4). As expected, both DPPH and FRAP values decreased significantly (*p* < 0.05) among all treatments with storage. The free radical scavenging effect of free/nanoencapsulated essential oils has been shown to have a concentration-dependent activity [42], so it naturally follows that as storage progressed, the concentration of bioactive compounds decreased and so did the anti-oxidant effect, as is evident by the increase in lipid oxidation parameters which also exhibited an increasing trend with storage. However, the decrease in radical scavenging activities was much less in T2 and T3, which were able to maintain higher anti-oxidant activities at the end of the storage period as compared to other treatments for reasons like the protection of bioactive compounds and their sustained release, as have been amply explained in the previous sections. Within each storage interval, the DPPH and FRAP values of all the treatments significantly (*p* < 0.05) differed from each other, with T2 and T3 exhibiting higher values as compared to other treatments. The higher anti-oxidant effect of nanoparticle emulsion treatments as compared to synthetic anti-oxidant treatment can also be explained by the former’s use in higher concentrations (500 ppm) as compared to BHT (maximum permissible limit of 200 ppm). The higher DPPH and FRAP values of T3 than T2 could be because of the higher total phenolic content (TPC) of ONPs than TNPs. This showed the positive correlation between TPC and radical scavenging activity. 

#### 3.2.6. Texture Profile Analysis

A significant (*p* < 0.05) decrease in all the texture attributes (firmness, consistency, cohesiveness and Work of cohesion) was observed in all the emulsion treatments including the control (Table 5), which might be attributed to the degradation of lipids and proteins as storage progressed, owing to different mechanisms like the oxidative, microbial and enzymatic degradation of lipids and proteins. However, T2 and T3 were more effective in maintaining texture attributes over successive storage days compared to other groups including control. The decrease in texture attributes with storage in different meat products has also been reported by various authors [43]. Similarly, Hakimian et al. [44], also observed a decrease in the texture parameters (hardness, cohesiveness, viscosity and adhesiveness) of mayonnaise treated with ZnO nanoparticles during refrigerated storage. Both lipids and proteins play a significant (*p* < 0.05) role in the texture properties of meat products. The results coincide with increasing fat and protein oxidation as well as an increase in the total plate count of emulsion treatments with storage. Some bacterial species like *Pseudomonas* are known to be proteolytic, thus negatively affecting the product’s texture [45]. Similarly, the lipolytic feature of certain microbes also diminishes the texture attributes of the product [46]. In addition, protein solubility, which plays an important part in maintaining the necessary network to bind fat and water, can be negatively affected by oxidative damage forming insoluble complexes by protein crosslinking, thus contributing adversely to the texture of the meat product [47]. The decrease in firmness, consistency and cohesiveness values is consistent with myofibrillar protein degradation, which plays a key role in maintaining emulsion stability and texture attributes, as has been sufficiently proven in several studies [48].

#### 3.2.7. Colour Profile Analysis

Surface colour is one of the most important visual features of meat and can have a significant impact on customer purchase decisions. This is partly due to customers associating meat colour with wholesomeness, freshness and eating quality. During refrigerated storage, the lightness (L*) and redness (a*) colour parameters decreased while yellowness (b*) values increased in all treatments including the control with the progress in the storage period (Table 6). The colour changes are particularly related to the oxidation status of the myoglobin pigment, which is the water-soluble protein responsible for meat colour. The valence state of iron in myoglobin determines meat colour via four chemical forms of myoglobin, viz. deoxymyoglobin (purplish-pink colour), oxymyoglobin (bright-red colour), carboxymyoglobin (stable bright-red colour) and metmyoglobin (dark-brown colour). It has been reported that the oxidation of myoglobin (light pink) to metmyoglobin (dark brown) causes a reduction in lightness scores in meat products [49]. The a* values provide an indication of redness, another important colour parameter for the analysis of meat. Higher a* values are related to the existence of oxymyoglobin (bright-red colour of meat), whereas low a* values are attributed to the formation of brown-colour metmyoglobin. Thus, the lowering of a* values during aerobic storage can be attributed to the oxidation of oxymyoglobin to metmyoglobin [50]. The b* values, which are related to yellowness, also increased with storage and are thought to be related to metmyoglobin formation [51]. This increase in b* values with aerobic storage has also been reported in various studies by different researchers [49]. The significantly (*p* < 0.05) higher L* and a* values and lower b* values in nanoencapsulated essential oil emulsion treatments (T2 and T3) can be attributed to the anti-oxidant and antimicrobial properties of essential oil nanoparticles [28]. The total colour change or colour change over a specific time period (ΔE) is a useful parameter to show the total colour differences among various treatments over time. The National Bureau of Standards describes colour differences (ΔE) as minor changes between colours that are perceptible by humans, defined as follows: 0–0.5 (trace); 0.5–1.5 (slight); 1.5–3.0 (noticeable); 3.0–6.0 (appreciable); 6.0–12.0 (much); and >12.0 (very much) [52]. According to these standards, the overall colour change was “much” in T0 and T1 as compared to T2, T3 and T4, in which it was only “appreciable”, thus coinciding well with the higher anti-oxidant potential of T2, T3 and T4 than T0 and T1. In addition, the ΔE values of T2 and T3 were significantly (*p* < 0.05) lower than other treatments including the control, thus signifying that the essential oil nanoparticles were able to preserve colour better than other treatments because of their significant (*p* < 0.05) anti-oxidant as well as antimicrobial potency.

### 3.3. Microbiological Quality

The microbiological parameters, viz. the total plate count, total psychrotrophic count, coliform count and yeast and mould count, showed an increasing trend in all the treatments including the control (Table 7), which could be due to the reason that meat being a rich source of nutrients, is an excellent medium for the growth of microbes. The total plate count surpassed 7 log cfu/g (considered the upper microbiological limit for good-quality fresh meat) in T0 and T4 on the 9th day of storage, in T1 on the 12th day of storage and in T2 and T3 on the 15th day of storage, thus signifying the antimicrobial potency of CNPs, TNPs and ONPs, which were able to restrict the TPC for a much longer time. No microbiological evaluation was performed in any of the treatments beyond the day that they reached the maximum permissible limit (MPL) of 7 log cfu/g. Similarly, T0 and T4 crossed the maximum psychrotrophic limit of 6 log cfu/g [53] on day 9, while in other treatments, the values were way below this limit. With regard to the coliform count, T0 and T4 surpassed 3 log cfu/g, the standard coliform limit [54], on day 9 and T1 on day 12. The coliform counts of all other treatments were well below the standard coliform limit. Although the coliform count also showed an increasing trend with storage, in T2 and T3, the counts showed a non-significant (*p* < 0.05) decrease from day 12 to day 15, which could be because of competitive inhibition by other spoilage bacteria, particularly pseudomonads, that show better adaptation to refrigeration temperatures [54]. A similar trend of a decrease in coliform counts was also reported by Kamkar et al. [55] in chicken emulsion and chicken breast fillets, respectively. The significantly (*p* < 0.05) lower microbial counts in the T1, T2 and T3 treatments might be because of the proven antimicrobial effect of essential oils along with chitosan. Thymol and carvacrol, the main antibacterial chemicals discovered in thyme and oregano essential oils, can disintegrate the outer membrane of Gram-negative bacteria, releasing lipopolysaccharide components and increasing the permeability of the adenosine triphosphate of the membrane, thereby changing its passive permeability [56]; meanwhile, in fungi these two phenolic compounds cause alterations in the hyphal morphology and hyphal aggregates, resulting in reduced hyphal diameters and lyses of the hyphal wall [57]. In addition, essential oils bind to ergosterol in fungal cell membranes, altering cell membrane permeability in addition to other effects like cell wall damage and the coagulation of cytoplasm [58].

### 3.4. Emulsion Stability

The total fluid released (TFR) is an important index of emulsion stability. A significant (*p* < 0.05) increase in TFR values (decrease in emulsion stability) was observed in all the treatments during refrigerated storage; however, T1, T2 and T3 had lower TFR values than T0 and T4 (Table 8). The lower TFR values (higher emulsion stability) of T1, T2 and T3 could be attributed to the higher water and fat binding capacity of chitosan [59]. Lipid oxidation (enzymatic or non-enzymatic) as well as the oxidative degradation and microbial decomposition of meat proteins causes a decrease in the emulsion stability of meat [60]. Most importantly, protein oxidation leads to the formation of protein carbonyls, which could affect the water-holding capacity (WHC) of muscle proteins through their involvement in the formation of inter- and intra-molecular crosslinks, which are known to reduce the WHC of meat [61]. As per Liu et al. [62], the loss of WHC in myofibrillar proteins is linked to the formation of intense crosslinking between proteins as a result of oxidative stress. The higher emulsion stability values (lower TFR values) in T2 and T3 on subsequent storage days may be attributed to the anti-oxidative and antimicrobial potential of the essential oil nanoparticles, which offered the emulsion treatments significant (*p* < 0.05) protection against oxidative and microbial degradation. Akhter et al. [19] found higher emulsion stability (lower TFR) values in emulsion treated with rosemary extract as compared to a control. Similarly, Verma et al. [60] found higher emulsion stability values in hydrolysate-treated emulsions as compared to a control and BHT and attributed the same to the functional properties of protein hydrolysates, which exhibited significant anti-oxidant and antimicrobial properties as well.

### 3.5. Sensory Evaluation

The sensory analysis of the meat emulsion was conducted by evaluating it on the basis of odour, colour, texture and overall acceptability scores (Table 9). The samples with a minimum score of 3.5, corresponding to the “acceptable score” as per the score card adopted, were considered to be acceptable for consumption. When consumers approach raw meat, the first thing they encounter is its aroma/odour. This initial olfactory experience can set the tone for their overall perception of the product and allows them to make quick judgments about whether they want to purchase and consume a particular meat product without further investigation. Foul or unpleasant odours can be an indication of spoilage (microbial contamination as well as oxidative deterioration), especially in meat emulsion, which is a raw, high-fat intermediate meat product susceptible to both oxidative and microbial degradation. In the current study, once a sample emitted any unpleasant odour, sensory evaluation was stopped, and the sample was not subjected to any further sensory evaluation for any other attribute. As storage progressed, a significant (*p* < 0.05) decrease was observed in all the sensory attributes, which coincided well with the results of the emulsion stability (%TFR), microbial and oxidation status of different emulsion treatments. Meat spoilage can be caused by oxidation products such as aldehydes, ketones, alcohols, esters, hydrocarbons and ammonia, as well as microbiological deterioration, as evidenced by off-flavour, off-odour, slime development and discoloration [63]. Based primarily on minimum acceptability scores of >3.5 being satisfactory, the storage life of aerobically packaged T0 (control) and T4 (positive control) meat emulsions was found to be 3 days, while that of T1 was found to be 6 days. T2 and T3 emulsions exhibited the maximum storage life of 9 days. The enhanced storage life of T2 and T3 corresponds well with the improved anti-oxidative and antimicrobial status of these treatments as imparted by thyme and oregano nanoparticles and correspond well with the improved sensory evaluation results as well as enhanced storage life of different nano-based treatments [31,64].

## 4. Conclusions

The use of thyme and oregano essential oil nanoparticles improved the quality and shelf life of meat emulsions during refrigerated storage. They outperformed the control and synthetic preservatives in terms of preserving physico-chemical, microbiological and sensory qualities. The nanoencapsulation of essential oils improved their anti-oxidant and antimicrobial efficacy, as indicated by decreased lipid and protein oxidation, increased radical scavenging capabilities, and better colour and texture retention of meat emulsion. The addition of thyme and oregano essential oil nanoparticles improved the emulsion durability. The sensory analysis validated these findings, as these groups maintained higher scores for odour, colour, texture and overall acceptability during the storage period. This study demonstrates the efficacy of nanoencapsulated essential oils as natural preservatives, providing a sustainable and safe alternative to synthetic preservatives for increasing the shelf life and quality of meat emulsion. Future research should look into environmental impacts, regulatory compliance and potential synergies with other natural preservatives to improve the commercial feasibility and global adoption of these natural nano-preservatives in the meat industry.

## Figures and Tables

**Table 1:** Treatment details of the experiment.

Treatments	Emulsion Type
T0	Control, containing no additives
T1	Incorporated with chitosan nanoparticles (CNPs) @ 500 ppm
T2	Incorporated with thyme essential oil nanoparticles (TNPs) @ 500 ppm
T3	Incorporated with oregano essential oil nanoparticles (ONPs) @ 500 ppm
T4	Positive control, incorporated with free butylated hydroxytoluene (BHT) @ 200 ppm

**Table 2:** pH of different treatments of meat emulsion during aerobic refrigerated storage.

Treatment	Storage Period (Days)					
	Day 0	Day 3	Day 6	Day 9	Day 12	Day 15
T0	5.98 ± 0.01 ^A^	6.16 ± 0.04 ^2B^	6.35 ± 0.03 ^2C^	6.54 ± 0.06 ^3D^	6.72 ± 0.07 ^3E^	6.91 ± 0.03 ^3F^
T1	5.94 ± 0.03 ^A^	6.05 ± 0.05 ^12A^	6.27 ± 0.07 ^2B^	6.36 ± 0.03 ^2BC^	6.53 ± 0.04 ^2C^	6.75 ± 0.08 ^2D^
T2	5.95 ± 0.04 ^A^	5.99 ± 0.04 ^1AB^	6.06 ± 0.05 ^1AB^	6.15 ± 0.04 ^1BC^	6.34 ± 0.07 ^1CD^	6.56 ± 0.07 ^1D^
T3	5.93 ± 0.05 ^A^	5.97 ± 0.04 ^1A^	6.04 ± 0.08 ^1A^	6.14 ± 0.08 ^1AB^	6.30 ± 0.06 ^1BC^	6.51 ± 0.05 ^1C^
T4	5.95 ± 0.07 ^A^	6.13 ± 0.06 ^12AB^	6.31 ± 0.05 ^2BC^	6.57 ± 0.09 ^3CD^	6.69 ± 0.05 ^3DE^	6.87 ± 0.06 ^3E^

Mean ± S.E. values with different superscripts column-wise (^1–3^) and row-wise (^A–F^) differ significantly (*p* < 0.05). T0: control emulsion; T1: emulsion treated with CNPs @ 500 ppm; T2: emulsion treated with TNPs @ 500 ppm; T3: emulsion treated with ONPs @ 500 ppm; T4: positive control emulsion @ 200 ppm.

**Table 3:** Peroxide value (meq/kg), TBARS (mg MDA/kg) and carbonyl content (nmol/mg protein) of different treatments of meat emulsion during aerobic refrigerated storage.

Treatment	Storage Period (Days)					
	Day 0	Day 3	Day 6	Day 9	Day 12	Day 15
**Peroxide Value (meq/kg)**						
T0	2.85 ± 0.04 ^3A^	4.83 ± 0.08 ^3B^	7.49 ± 0.17 ^3D^	10.66 ± 0.12 ^4F^	8.23 ± 0.02 ^4E^	6.89 ± 0.23 ^4C^
T1	1.85 ± 0.02 ^2A^	3.39 ± 0.03 ^2B^	5.26 ± 0.02 ^2C^	8.16 ± 0.01 ^3E^	7.64 ± 0.12 ^3D^	5.30 ± 0.03 ^3C^
T2	1.58 ± 0.01 ^1A^	2.96 ± 0.04 ^1B^	4.19 ± 0.01 ^1C^	5.56 ± 0.02 ^1F^	5.39 ± 0.09 ^1E^	4.84 ± 0.03 ^12D^
T3	1.56 ± 0.01 ^1A^	2.93 ± 0.02 ^1B^	4.16 ± 0.01 ^1C^	5.52 ± 0.03 ^1F^	5.30 ± 0.10 ^1E^	4.81 ± 0.02 ^1D^
T4	1.54 ± 0.02 ^1A^	2.92 ± 0.02 ^1B^	4.23 ± 0.02 ^1C^	6.32 ± 0.06 ^2F^	6.08 ± 0.02 ^2E^	5.17 ± 0.01 ^23D^
**TBARS (mg MDA/kg)**						
T0	0.37 ± 0.01 ^3A^	0.55 ± 0.02 ^3B^	0.72 ± 0.02 ^3C^	0.92 ± 0.06 ^4D^	1.13 ± 0.05 ^4E^	1.25 ± 0.05 ^4E^
T1	0.24 ± 0.01 ^2A^	0.43 ± 0.01 ^2B^	0.58 ± 0.03 ^2C^	0.74 ± 0.01 ^3D^	0.92 ± 0.01 ^3E^	1.04 ± 0.01 ^3F^
T2	0.14 ± 0.01 ^1A^	0.26 ± 0.02 ^1B^	0.37 ± 0.02 ^1C^	0.46 ± 0.01 ^1D^	0.66 ± 0.01 ^1E^	0.85 ± 0.01 ^1F^
T3	0.13 ± 0.01 ^1A^	0.24 ± 0.01 ^1B^	0.34 ± 0.01 ^1C^	0.42 ± 0.01 ^1D^	0.64 ± 0.01 ^1E^	0.83 ± 0.01 ^1F^
T4	0.12 ± 0.01 ^1A^	0.22 ± 0.01 ^1B^	0.36 ± 0.01 ^1C^	0.57 ± 0.01 ^2D^	0.76 ± 0.02 ^2E^	0.93 ± 0.01 ^2F^
**Carbonyl Content (nmol/mg protein)**						
T0	0.96 ± 0.01 ^3A^	1.25 ± 0.01 ^3B^	1.56 ± 0.01 ^3C^	2.66 ± 0.02 ^4D^	3.44 ± 0.11 ^4E^	4.49 ± 0.02 ^4F^
T1	0.85 ± 0.02 ^2A^	1.16 ± 0.02 ^2B^	1.34 ± 0.02 ^2C^	1.66 ± 0.01 ^3D^	1.95 ± 0.01 ^3E^	2.85 ± 0.02 ^3F^
T2	0.75 ± 0.01 ^1A^	0.94 ± 0.01 ^1B^	1.14 ± 0.01 ^1C^	1.34 ± 0.01 ^1D^	1.56 ± 0.01 ^1E^	1.74 ± 0.01 ^1F^
T3	0.73 ± 0.01 ^1A^	0.93 ± 0.01 ^1B^	1.13 ± 0.01 ^1C^	1.33 ± 0.01 ^1D^	1.52 ± 0.02 ^1E^	1.73 ± 0.01 ^1F^
T4	0.75 ± 0.01 ^1A^	0.95 ± 0.01 ^1B^	1.17 ± 0.01 ^1C^	1.47 ± 0.01 ^2D^	1.75 ± 0.01 ^2E^	1.96 ± 0.01 ^2F^

Mean ± S.E. values with different superscripts column-wise (^1–4^) and row-wise (^A–F^) differ significantly (*p* < 0.05). T0: control emulsion; T1: emulsion treated with CNPs @ 500 ppm; T2: emulsion treated with TNPs @ 500 ppm; T3: emulsion treated with ONPs @ 500 ppm; T4: positive control emulsion @ 200 ppm. TBARS: Thiobarbituric acid reactive substances.

**Table 4:** DPPH and FRAP of different treatments of meat emulsion during aerobic refrigerated storage.

Treatment	Storage Period (Days)					
	Day 0	Day 3	Day 6	Day 9	Day 12	Day 15
**DPPH (% inhibition)**						
T0	17.14 ± 0.10 ^1F^	13.15 ± 0.11 ^1E^	11.14 ± 0.13 ^1D^	8.41 ± 0.22 ^1C^	6.20 ± 0.11 ^1B^	4.33 ± 0.12 ^1A^
T1	30.34 ± 0.52 ^2F^	27.26 ± 0.28 ^2E^	24.06 ± 0.15 ^2D^	20.08 ± 0.12 ^2C^	17.40 ± 0.08 ^2B^	15.07 ± 0.21 ^1A^
T2	61.08 ± 0.18 ^4F^	57.19 ± 0.05 ^4E^	54.19 ± 0.31 ^4D^	51.04 ± 0.21 ^4C^	47.08 ± 0.10 ^4B^	43.20 ± 0.04 ^4A^
T3	64.46 ± 0.44 ^5F^	61.09 ± 0.40 ^5E^	57.16 ± 0.20 ^5D^	55.14 ± 0.28 ^5C^	51.09 ± 0.21 ^5B^	46.20 ± 0.35 ^5A^
T4	49.70 ± 0.24 ^3F^	45.41 ± 0.18 ^3E^	41.75 ± 0.23 ^3D^	36.72 ± 0.47 ^3C^	33.12 ± 0.19 ^3B^	30.08 ± 0.11 ^3A^
**FRAP (mM Fe^2+^ Eq)**						
T0	11.56 ± 0.17 ^1F^	9.39 ± 0.14 ^1E^	7.38 ± 0.08 ^1D^	6.50 ± 0.12 ^1C^	5.53 ± 0.16 ^1B^	4.51 ± 0.12 ^1A^
T1	18.65 ± 0.17 ^2F^	15.52 ± 0.19 ^2E^	13.62 ± 0.15 ^2D^	12.22 ± 0.03 ^2C^	10.55 ± 0.15 ^2B^	9.62 ± 0.14 ^2A^
T2	32.50 ± 0.09 ^4F^	29.62 ± 0.18 ^4E^	26.44 ± 0.16 ^4D^	24.64 ± 0.12 ^4C^	23.48 ± 0.15 ^4B^	20.67 ± 0.12 ^4A^
T3	35.55 ± 0.19 ^5F^	33.47 ± 0.13 ^5E^	30.43 ± 0.06 ^5D^	27.67 ± 0.26 ^5C^	25.53 ± 0.17 ^5B^	23.29 ± 0.07 ^5A^
T4	22.41 ± 0.14 ^3F^	19.52 ± 0.10 ^3E^	17.40 ± 0.09 ^3D^	14.63 ± 0.20 ^3C^	12.31 ± 0.04 ^3B^	10.25 ± 0.06 ^3A^

Mean ± S.E. values with different superscripts column-wise (1–5) and row-wise (A–F) differ significantly (*p* < 0.05). T0: control emulsion; T1: emulsion treated with CNPs @ 500 ppm; T2: emulsion treated with TNPs @ 500 ppm; T3: emulsion treated with ONPs @ 500 ppm; T4: positive control emulsion @ 200 ppm. DPPH: 2, 2 diphenyl-1-picryl hydrazyl; FRAP: Ferric Reducing Anti-Oxidant Power.

**Table 5:** Texture attributes of different treatments of meat emulsion during aerobic refrigerated storage.

Treatment	Storage Period (Days)					
	Firmness (N)			Consistency (N.s)		
	Day 0	Day 7	Day 15	Day 0	Day 7	Day 15
T0	52.37 ± 0.29 ^C^	39.12 ± 0.80 ^1B^	30.16 ± 0.39 ^1A^	2123.36 ± 68.22^C^	1025.24 ± 26.88 ^1B^	568.81 ± 52.87 ^A^
T1	54.85 ± 0.98 ^C^	45.10 ± 0.36 ^3B^	33.71 ± 0.77 ^23A^	2302.07 ± 310.72^B^	1724.13 ± 73.19 ^2B^	695.35 ± 59.20 ^A^
T2	54.49 ± 0.66 ^C^	47.49 ± 0.81 ^4B^	35.23 ± 0.85 ^34A^	2164.37 ± 91.07 ^B^	1871.04 ± 46.93 ^23B^	729.68 ± 58.72 ^A^
T3	54.37 ± 0.54 ^C^	47.54 ± 0.47 ^4B^	36.42 ± 0.78 ^4A^	2108.06 ± 256.30^B^	1998.78 ± 122.74 ^3B^	750.59 ± 48.00 ^A^
T4	53.12 ± 0.98 ^C^	42.77 ± 0.32 ^2B^	31.67 ± 0.87 ^12A^	2108.85 ± 101.78 ^C^	1042.61 ± 24.61^1B^	570.68 ± 54.79 ^A^
**Treatment**	**Cohesiveness (N)**			**Work of Cohesion (N.s)**		
	**Day 0**	**Day 7**	**Day 15**	**Day 0**	**Day 7**	**Day 15**
T0	(−)45.73 ± 0.66 ^C^	(−)34.18 ± 0.66 ^1B^	(−)16.63 ± 0.80 ^1A^	(−)37.01 ± 0.16 ^C^	(−)12.21 ± 0.84 ^1B^	(−)5.46 ± 0.15 ^1A^
T1	(−)47.71 ± 1.37 ^C^	(−)42.01 ± 0.28 ^3B^	(−)19.74 ± 1.49 ^2A^	(−)37.81 ± 0.71 ^C^	(−)16.48 ± 0.84 ^23B^	(−)8.37 ± 0.10 ^2A^
T2	(−)46.21 ± 0.88 ^C^	(−)42.25 ± 0.23 ^3B^	(−)20.84 ± 0.36 ^2A^	(−)37.90 ± 0.40 ^C^	(−)16.50 ± 0.64 ^23B^	(−)9.31 ± 0.28 ^3A^
T3	(−)47.63 ± 0.89 ^C^	(−)41.32 ± 0.30 ^3B^	(−)21.11 ± 0.17 ^2A^	(−)37.47 ± 0.53 ^C^	(−)16.88 ± 0.71 ^3B^	(−)9.11 ± 0.02 ^3A^
T4	(−)46.21 ± 0.48 ^C^	(−)35.88 ± 0.85 ^2B^	(−)16.99 ± 0.38 ^1A^	(−)36.37 ± 0.84 ^C^	(−)13.93 ± 0.96 ^12B^	(−)5.59 ± 0.22 ^1A^

Mean ± S.E. values with different superscripts column-wise (1–4) and row-wise (A–C) differ significantly (*p* < 0.05). T0: control emulsion; T1: emulsion treated with CNPs @ 500 ppm; T2: emulsion treated with TNPs @ 500 ppm; T3: emulsion treated with ONPs @ 500 ppm; T4: positive control emulsion @ 200 ppm. The negative (−) sign of the above values indicates the back extrusion force (Pull) against gravity; it does not change the absolute value of a number.

**Table 6:** Colour attributes of different treatments of meat emulsion during aerobic refrigerated storage.

Treatment	Storage Period (Days)					
	Lightness (L*)			Redness (a*)		
	Day 0	Day 7	Day 15	Day 0	Day 7	Day 15
T0	52.49 ± 0.09 ^C^	48.44 ± 0.12 ^1B^	45.20 ± 0.03 ^1A^	12.29 ± 0.09 ^C^	9.33 ± 0.06 ^1B^	8.12 ± 0.19 ^1A^
T1	52.57 ± 0.08 ^C^	50.39 ± 0.06 ^2B^	47.39 ± 0.07 ^2A^	12.50 ± 0.07 ^C^	10.65 ± 0.01 ^2B^	8.57 ± 0.08 ^2A^
T2	52.39 ± 0.12 ^C^	51.52 ± 0.07 ^3B^	49.37 ± 0.08 ^4A^	12.43 ± 0.12 ^C^	11.06 ± 0.01 ^4B^	9.43 ± 0.01 ^4A^
T3	52.31 ± 0.09 ^C^	51.56 ± 0.06 ^3B^	49.19 ± 0.03 ^4A^	12.34 ± 0.09 ^C^	11.84 ± 0.01 ^5B^	10.70 ± 0.11 ^5A^
T4	52.45 ± 0.12 ^C^	51.38 ± 0.05 ^3B^	48.44 ± 0.08 ^3A^	12.50 ± 0.04 ^C^	10.93 ± 0.01 ^3B^	9.09 ± 0.04 ^3A^
**Treatment**	Yellowness (b*)			**Overall Colour Change (ΔE)**		
	**Day 0**	**Day 7**	**Day 15**	**Day (0–15)**		
T0	14.48 ± 0.10 ^A^	15.77 ± 0.31 ^3B^	17.61 ± 0.29 ^4C^	8.99 ± 0.15 ^5^		
T1	14.44 ± 0.08 ^A^	15.10 ± 0.17 ^2B^	16.90 ± 0.20 ^3C^	6.99 ± 0.08 ^4^		
T2	14.37 ± 0.10 ^A^	14.42 ± 0.09 ^1A^	15.36 ± 0.25 ^1B^	4.44 ± 0.17 ^2^		
T3	14.29 ± 0.03 ^A^	14.36 ± 0.08 ^1A^	15.25 ± 0.04 ^1B^	3.68 ± 0.11 ^1^		
T4	14.40 ± 0.06 ^A^	14.65 ± 0.16 ^12A^	16.14 ± 0.32 ^2B^	5.58 ± 0.15 ^3^		

Mean ± S.E. values with different superscripts column-wise (^1–5^) and row-wise (^A–C^) differ significantly (*p* < 0.05). T0: control emulsion; T1: emulsion treated with CNPs @ 500 ppm; T2: emulsion treated with TNPs @ 500 ppm; T3: emulsion treated with ONPs @ 500 ppm; T4: positive control emulsion @ 200 ppm.

**Table 7:** Microbiological quality of different treatments of meat emulsion during aerobic refrigerated storage.

Treatment	Storage Period (Days)					
	Day 0	Day 3	Day 6	Day 9	Day 12	Day 15
**Total Plate Count (log_10_CFU/g)**						
T0	4.10 ± 0.14 ^A^	5.68 ± 0.08 ^2B^	6.43 ± 0.05 ^3C^	7.21 ± 0.20 ^3D^	NP	NP
T1	3.84 ± 0.15 ^A^	4.14 ± 0.04 ^1A^	5.29 ± 0.23 ^2B^	6.62 ± 0.10 ^2C^	7.10 ± 0.07 ^2D^	NP
T2	3.75 ± 0.07 ^A^	3.91 ± 0.10 ^1A^	4.84 ± 0.09 ^1B^	5.68 ± 0.15 ^1C^	6.32 ± 0.07 ^1D^	7.14 ± 0.01 ^E^
T3	3.71 ± 0.15 ^A^	3.87 ± 0.26 ^1A^	4.78 ± 0.05 ^1B^	5.72 ± 0.15 ^1C^	6.38 ± 0.11 ^1D^	7.12 ± 0.01 ^E^
T4	4.13 ± 0.08 ^A^	5.71 ± 0.04 ^2B^	6.49 ± 0.12 ^3C^	7.28 ± 0.10 ^3D^	NP	NP
**Total Psychrotrophic Count (log_10_CFU/g)**						
T0	3.65 ± 0.04 ^2A^	4.69 ± 0.06 ^3B^	5.84 ± 0.04 ^3C^	6.58 ± 0.08 ^3D^	NP	NP
T1	3.48 ± 0.03 ^1A^	3.89 ± 0.10 ^2B^	4.14 ± 0.02 ^2C^	5.23 ± 0.08 ^2D^	5.98 ± 0.06 ^2E^	NP
T2	3.36 ± 0.08 ^1A^	3.48 ± 0.05 ^1A^	3.91 ± 0.04 ^1B^	4.55 ± 0.05 ^1C^	4.79 ± 0.06 ^1D^	5.24 ± 0.07 ^E^
T3	3.32 ± 0.04 ^1A^	3.42 ± 0.05 ^1A^	3.86 ± 0.09 ^1B^	4.49 ± 0.04 ^1C^	4.72 ± 0.07 ^1D^	5.19 ± 0.03 ^E^
T4	3.73 ± 0.04 ^2A^	4.74 ± 0.11 ^3B^	5.89 ± 0.03 ^3C^	6.62 ± 0.07 ^3D^	NP	NP
**Coliform Count (log_10_CFU/g)**						
T0	2.59 ± 0.11 ^2A^	2.78 ± 0.13 ^2AB^	2.95 ± 0.06 ^2BC^	3.12 ± 0.08 ^2C^	NP	NP
T1	2.38 ± 0.08 ^12A^	2.47 ± 0.10 ^12AB^	2.71 ± 0.03 ^12AB^	2.83 ± 0.02 ^1AB^	3.08 ± 0.11 ^2B^	NP
T2	2.26 ± 0.07 ^1A^	2.35 ± 0.12 ^1AB^	2.48 ± 0.23 ^1AB^	2.66 ± 0.11 ^1AB^	2.86 ± 0.06 ^1B^	2.74 ± 0.08 ^AB^
T3	2.22 ± 0.04 ^1A^	2.30 ± 0.10 ^1AB^	2.42 ± 0.06 ^1AB^	2.61 ± 0.07 ^1AB^	2.81 ± 0.06 ^1B^	2.68 ± 0.09 ^AB^
T4	2.54 ± 0.08 ^2A^	2.73 ± 0.08 ^2AB^	2.90 ± 0.10 ^2AB^	3.19 ± 0.04 ^2B^	NP	NP
**Yeast and Mould Count (log_10_CFU/g)**						
T0	1.53 ± 0.15 ^A^	2.32 ± 0.03 ^2B^	2.43 ± 0.10 ^2BC^	2.65 ± 0.09 ^2C^	NP	NP
T1	1.45 ± 0.15 ^A^	1.93 ± 0.07 ^1B^	2.20 ± 0.06 ^1C^	2.38 ± 0.05 ^1CD^	2.49 ± 0.12 ^1CD^	NP
T2	1.36 ± 0.03 ^A^	1.84 ± 0.05 ^1B^	2.16 ± 0.08 ^1C^	2.27 ± 0.04 ^1CD^	2.37 ± 0.03 ^1D^	2.42 ± 0.04 ^1D^
T3	1.32 ± 0.02 ^A^	1.81 ± 0.03 ^1B^	2.10 ± 0.09 ^1C^	2.23 ± 0.04 ^1CD^	2.31 ± 0.02 ^1DE^	2.39 ± 0.04 ^1E^
T4	1.57 ± 0.08 ^A^	2.38 ± 0.05 ^2B^	2.47 ± 0.04 ^2B^	2.72 ± 0.05 ^2C^	NP	NP

Mean ± S.E. values with different superscripts column-wise (^1–4^) and row-wise (^A–E^) differ significantly (*p* < 0.05). T0: control emulsion; T1: emulsion treated with CNPs @ 500 ppm; T2: emulsion treated with TNPs @ 500 ppm; T3: emulsion treated with ONPs @ 500 ppm; T4: positive control emulsion @ 200 ppm. NP = evaluation not performed (since the concerned samples emanated a foul odour with TPC above 7 log (MPL), further microbiological examination of these particular samples was not performed).

**Table 8:** Emulsion stability (%TFR) of different treatments of meat emulsion during aerobic refrigerated storage.

Treatment	Storage Period (Days)		
	Day 0	Day 7	Day 15
T0	4.79 ± 0.30 ^2A^	10.53 ± 0.14 ^3B^	21.37 ± 0.37 ^3C^
T1	4.21 ± 0.21 ^1A^	8.43 ± 0.11 ^2B^	18.53 ± 0.44 ^2C^
T2	4.26 ± 0.06 ^1A^	7.53 ± 0.08 ^1B^	15.75 ± 0.13 ^1C^
T3	4.24 ± 0.05 ^1A^	7.49 ± 0.05 ^1B^	14.66 ± 0.05 ^1C^
T4	4.85 ± 0.12 ^2A^	10.80 ± 0.28 ^3B^	21.81 ± 0.62 ^3C^

Mean ± S.E. values with different superscripts column-wise (^1–3^) and row-wise (^A–C^) differ significantly (*p* < 0.05). T0: control emulsion; T1: emulsion treated with CNPs @ 500 ppm; T2: emulsion treated with TNPs @ 500 ppm; T3: emulsion treated with ONPs @ 500 ppm; T4: positive control emulsion @ 200 ppm.

**Table 9:** Sensory evaluation of different treatments of meat emulsion during aerobic refrigerated storage.

Treatment	Storage Period (Days)					
	Day 0	Day 3	Day 6	Day 9	Day 12	Day 15
**Odour**						
T0	4.77 ± 0.13 ^D^	4.20 ± 0.13 ^1C^	2.41 ± 0.12 ^1B^	1.00 ± 0.00 ^1A^	NP	NP
T1	4.75 ± 0.13 ^E^	4.42 ± 0.12 ^2D^	3.67 ± 0.13 ^3C^	2.54 ± 0.13 ^2B^	1.00 ± 0.00 ^1A^	NP
T2	4.73 ± 0.14 ^F^	4.58 ± 0.14 ^3E^	3.83 ± 0.12 ^4D^	3.50 ± 0.13 ^3C^	2.53 ± 0.13 ^2B^	1.00 ± 0.00 ^A^
T3	4.78 ± 0.15 ^F^	4.53 ± 0.15 ^3E^	3.89 ± 0.11 ^4D^	3.58 ± 0.15 ^3C^	2.59 ± 0.15 ^2B^	1.00 ± 0.00 ^A^
T4	4.71 ± 0.14 ^D^	4.25 ± 0.15 ^1C^	2.49 ± 0.14 ^2B^	1.00 ± 0.00 ^1A^	NP	NP
**Colour**						
T0	5.00 ± 0.00 ^C^	4.52 ± 0.12 ^B^	3.21 ± 0.12 ^1A^	NP	NP	NP
T1	5.00 ± 0.00 ^D^	4.51 ± 0.12 ^C^	3.67 ± 0.13 ^2B^	3.28 ± 0.13 ^1A^	NP	NP
T2	5.00 ± 0.00 ^E^	4.54 ± 0.12 ^D^	3.83 ± 0.13 ^3C^	3.71 ± 0.15 ^2B^	3.55 ± 0.13 ^A^	NP
T3	5.00 ± 0.00 ^E^	4.56 ± 0.13 ^D^	3.92 ± 0.15 ^3C^	3.75 ± 0.13 ^2B^	3.58 ± 0.15 ^A^	NP
T4	5.00 ± 0.00 ^C^	4.50 ± 0.13 ^B^	3.85 ± 0.13 ^3A^	NP	NP	NP
**Texture**						
T0	4.75 ± 0.14 ^C^	4.63 ± 0.13 ^B^	3.12 ± 0.12 ^1A^	NP	NP	NP
T1	4.78 ± 0.14 ^D^	4.65 ± 0.13 ^C^	3.67 ± 0.15 ^2B^	3.15 ± 0.13 ^1A^	NP	NP
T2	4.72 ± 0.13 ^D^	4.67 ± 0.13 ^D^	3.75 ± 0.13 ^3C^	3.58 ± 0.12 ^2B^	3.24 ± 0.14 ^A^	NP
T3	4.74 ± 0.13 ^D^	4.69 ± 0.12 ^D^	3.71 ± 0.14 ^23C^	3.55 ± 0.13 ^2B^	3.27 ± 0.14 ^A^	NP
T4	4.75 ± 0.14 ^C^	4.62 ± 0.13 ^B^	3.17 ± 0.15 ^1A^	NP	NP	NP
**Overall Acceptability**						
T0	4.82 ± 0.13 ^D^	4.42 ± 0.12 ^1C^	2.62 ± 0.11 ^1B^	1.00 ± 0.00 ^1A^	NP	NP
T1	4.84 ± 0.12 ^E^	4.51 ± 0.14 ^2D^	3.65 ± 0.13 ^3C^	2.86 ± 0.14 ^2B^	1.00 ± 0.00 ^1A^	NP
T2	4.80 ± 0.13 ^F^	4.59 ± 0.15 ^23E^	3.80 ± 0.15 ^4D^	3.56 ± 0.14 ^3C^	2.91 ± 0.11 ^2B^	1.00 ± 0.00 ^A^
T3	4.83 ± 0.15 ^F^	4.62 ± 0.15 ^3E^	3.85 ± 0.12 ^4D^	3.61 ± 0.15 ^3C^	2.95 ± 0.14 ^2B^	1.00 ± 0.00 ^A^
T4	4.81 ± 0.15 ^D^	4.40 ± 0.15 ^1C^	2.83 ± 0.14 ^2B^	1.00 ± 0.00 ^1A^	NP	NP

Mean ± S.E. values with different superscripts column-wise (^1–4^) and row-wise (^A–F^) differ significantly (*p* < 0.05). T0: control emulsion; T1: emulsion treated with CNPs @ 500 ppm; T2: emulsion treated with TNPs @ 500 ppm; T3: emulsion treated with ONPs @ 500 ppm; T4: positive control emulsion @ 200 ppm. NP = evaluation not performed as the concerned samples were spoiled.

## Data Availability

The original contributions presented in the study are included in the article, further inquiries can be directed to the corresponding author.
